# Development of a Regenerative Peripheral Nerve Interface for Control of a Neuroprosthetic Limb

**DOI:** 10.1155/2016/5726730

**Published:** 2016-05-17

**Authors:** Melanie G. Urbanchek, Theodore A. Kung, Christopher M. Frost, David C. Martin, Lisa M. Larkin, Adi Wollstein, Paul S. Cederna

**Affiliations:** ^1^Section of Plastic and Reconstructive Surgery, Department of Surgery, University of Michigan Health System, Ann Arbor, MI 48109-5463, USA; ^2^Department of Materials Science and Engineering, University of Delaware, Newark, DE 19716-1501, USA; ^3^Department of Molecular and Integrative Physiology, University of Michigan, Ann Arbor, MI 48109-2200, USA; ^4^Biomedical Engineering, College of Engineering, University of Michigan, Ann Arbor, MI 48109-2110, USA

## Abstract

*Background*. The purpose of this experiment was to develop a peripheral nerve interface using cultured myoblasts within a scaffold to provide a biologically stable interface while providing signal amplification for neuroprosthetic control and preventing neuroma formation.* Methods*. A Regenerative Peripheral Nerve Interface (RPNI) composed of a scaffold and cultured myoblasts was implanted on the end of a divided peroneal nerve in rats (*n* = 25). The scaffold material consisted of either silicone mesh, acellular muscle, or acellular muscle with chemically polymerized poly(3,4-ethylenedioxythiophene) conductive polymer. Average implantation time was 93 days. Electrophysiological tests were performed at endpoint to determine RPNI viability and ability to transduce neural signals. Tissue samples were examined using both light microscopy and immunohistochemistry.* Results*. All implanted RPNIs, regardless of scaffold type, remained viable and displayed robust vascularity. Electromyographic activity and stimulated compound muscle action potentials were successfully recorded from all RPNIs. Physiologic efferent motor action potentials were detected from RPNIs in response to sensory foot stimulation. Histology and transmission electron microscopy revealed mature muscle fibers, axonal regeneration without neuroma formation, neovascularization, and synaptogenesis. Desmin staining confirmed the preservation and maturation of myoblasts within the RPNIs.* Conclusions*. RPNI demonstrates significant myoblast maturation, innervation, and vascularization without neuroma formation.

## 1. Introduction

Breakthroughs in robotic technology have facilitated the advent of upper extremity prosthetic devices which have the capability to emulate the functions of a native extremity. However, realization of the full potential of these devices has been hindered by the lack of an optimal interface between the patient and the artificial limb. This crucial interface must permit reliable transmission of both efferent motor commands and afferent sensory signals of sufficient amplitude to be detectable above the inherent electrical noise. One of the more popular strategies to achieve prosthetic control involves a variety of experimental intraneural or epineural electrodes placed directly within or on the epineurial surface of peripheral nerves within the residual limb [1]. This technique is particularly attractive because a significant amount of axonal sorting and organization occurs within peripheral nerves; therefore, directly interfacing with residual peripheral nerves provides greatly increased signal specificity as compared to other types of control systems including brain interfaces. However, while many types of peripheral nerve interfaces (PNIs) have been studied and successfully utilized to transduce efferent motor action potentials, they are limited by their lack of long-term stability. The major design concern is to provide a sufficiently robust interface capable of detecting physiologic action potentials while limiting the axonal damage and foreign body reaction which subsequently leads to loss of signal fidelity [2]. In addition, a relatively unrecognized limitation with existing peripheral nerve interfaces is the inability to control neuroma formation in the residual limb.

The ideal PNI possesses a number of attributes which will consistently provide high-fidelity control of a neuroprosthetic device over a long period of time. The highly conductive interface should provide amplification and stable transmission of relatively low-amplitude nerve signals in order to provide fine motor control. It should promote integration with surrounding tissues to minimize the inevitable scarring and encapsulation which result in signal degradation over time. The PNI should be designed to avoid iatrogenic axonal damage within the peripheral nerve either at the time of implantation or from chronic micromotion. Furthermore, the ideal PNI should mitigate neuroma formation which can not only lead to pain but can also lead to signal interference from misdirected axons and inappropriately low depolarization potentials.

These attributes were carefully considered when conceptualizing the Regenerative Peripheral Nerve Interface (RPNI), a novel experimental nerve interface designed for long-term, stable integration with transected peripheral nerves in a residual limb. In this study, the RPNI was surgically constructed by inserting the distal end of a divided peripheral nerve into a cylindrical scaffold that was populated with cultured myoblasts. Maturation and innervation of the myoblasts within the RPNI provide amplification of neural signals and mitigate neuroma formation. To increase the conductivity of the RPNI construct, an electroconductive polymer can be applied to the scaffold material prior to implantation. This paper details the development of the RPNI and describes the proof-of-concept experiments performed to demonstrate its potential as an effective interface between divided peripheral nerves and neuroprosthetic devices.

## 2. Methods

All animal care and operative procedures were conducted in accordance with the Guide for the Care and Use of Laboratory Animals [3]. A single RPNI was constructed within the left thigh in each of 25 male F344 rats weighing between 300 and 400 grams (Charles River, Wilmington, MA). Each RPNI consisted of (1) a scaffold material; (2) cultured autogenous myoblasts; (3) the distal end of the divided peroneal nerve; and, in one experimental group, (4) chemically polymerized poly(3,4-ethylenedioxythiophene) (PEDOT) conductive polymer. Three study groups were established based on the scaffold material used to create the RPNI: (1) silicone mesh (*n* = 9), (2) acellular muscle (*n* = 10), and (3) acellular muscle with polymerized conductive polymer (*n* = 6). Silicone mesh and acellular muscle were chosen because both provide a sufficient amount of pliability and permeability while at the same time possessing enough durability to serve as a stable construct for implantation of cultured myocytes. PEDOT was chosen to determine if a conductive polymer polymerized onto the acellular muscle could be used in the setting of an RPNI.

Cell harvesting and culture were performed as previously described [4]. Soleus muscle myoblasts from isogenic female rats were grown in culture in 35 mm plates with growth medium consisting of 400 mL of HAMF-12 nutrient mixture (GibcoBRL) with 100 mL of fetal bovine serum (GibcoBRL) and 100 units/mL of Penicillin G (SIGMA). Serial passage technique was used to isolate and maximize the myoblasts population within the growth plate. Growth medium was replaced every 48 hours after cell plating. The cells were allowed to mature until 75% confluence was reached. At that time, the growth medium was replaced with differentiation medium consisting of 465 mL of DMEM (GibcoBRL) with 35 mL horse serum (GibcoBRL) and 100 units/mL Penicillin G. Differentiation medium induced formation of multinucleated myotubes and after a total culture period of 13 to 17 days the myotubes were ready. Myotubes were considered ready for implantation into the RPNI when the monolayers of myoblast cells were contracting.

Each RPNI was surgically fabricated in the same fashion and allowed to mature in vivo. After using pentobarbital sodium (50 mg/kg) to achieve anesthesia, a longitudinal incision was made on the lateral aspect of the left hind limb. Intramuscular dissection proceeded through the biceps femoris muscle until the common peroneal and tibial nerves were encountered within the midthigh. Meticulous dissection of the common peroneal nerve was performed distally under the operating microscope to the level where it enters the lateral compartment of the lower extremity. A 2 cm length of common peroneal nerve was resected just proximal to the lateral compartment of the lower limb.

**Figure 1:**
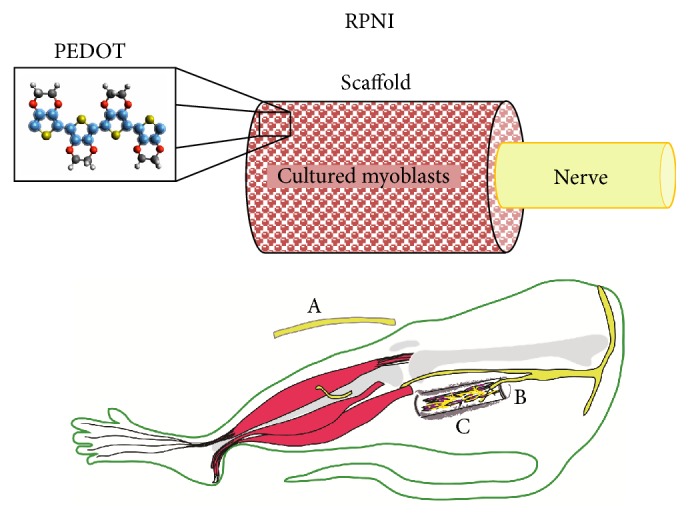
Schematic drawing of a Regenerative Peripheral Nerve Interface (RPNI) which is constructed using scaffold material consisting of either silicone mesh, acellular muscle, or acellular muscle with PEDOT conductive polymer. In this example, the end of the peripheral nerve is wrapped by acellular muscle with PEDOT and the construct is populated with cultured myoblasts. (Below) A 2 cm section of the distal common peroneal nerve is removed (A) and the residual nerve (B) is implanted into the RPNI (C) for a minimum of 2 months.

In the silicone mesh group (SM), a 15 × 10 mm piece of 0.5 mm thick silicone elastomer was cut to size and was manually meshed using a 1 mm punch biopsy tool to increase permeability, nutrient diffusion, and vascular ingrowth. The silicone was positioned at the distal end of the divided peroneal nerve. Several interrupted 9-0 nylon microsutures were placed through the epineurium and into the scaffold to secure the nerve with approximately 5 mm of overlap between the distal peroneal nerve and the proximal end of the scaffold. The scaffold was then wrapped into the shape of a cylinder and secured with suture. Two 35 mm plates of myotubes (3 million myoblasts by direct counting technique at plating day 7) were gently removed from the culture dish and transferred to each tube. The ends of the scaffold were closed using interrupted 7-0 Prolene sutures (Figure 1). In the acellular muscle group (AM), the scaffold material was prepared using previously described techniques to remove all cellular elements from mouse abdominal wall muscle specimens [5]. The resultant acellular muscle was then used to create an RPNI at the end of the peroneal nerve in the same manner as the silicone mesh. To investigate if the conductivity of the RPNI could be enhanced, PEDOT electroconductive polymer was chemically polymerized onto the acellular muscle scaffold in a separate group of 6 rats (AM+PEDOT) [6]. PEDOT is a biocompatible substance conducive to polymerization with biologic tissues and has been shown to reduce charge density and lower impedance in other experimental settings by increasing surface area [7–9]. After RPNI implantation, all surgical wounds were closed in a layered fashion and rats were allowed to recover for a minimum of two months.

### 2.1. Electrophysiological Testing

**Figure 2:**
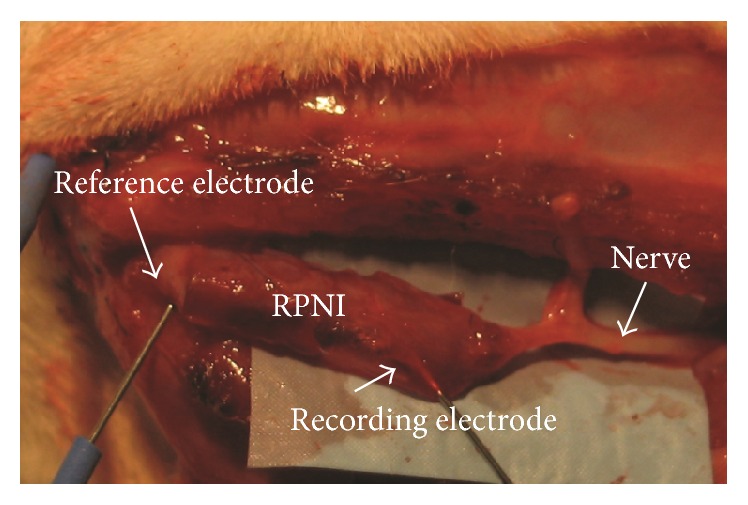
In situ image of Regenerative Peripheral Nerve Interface (RPNI), 4 months after implantation. In this example, 2 × 35 mm plates of myoblasts at culture day 14 were deposited on a one-layer thick sheet (2 cm long) acellular muscle. The common peroneal was transected, a 2 cm length was discarded, and the proximal residual end was tacked to the acellular muscle. The acellular muscle was rolled lengthwise to contain the myoblasts and maintain contact with the transected peroneal nerve. Evoked compound muscle action potential recording with stimulating electrode positioned on the peroneal nerve was 90 *μ*V peak-to-peak.

At the time of sacrifice, rats were anesthetized and the surgical site was reopened. The RPNI and proximal common peroneal nerve were carefully dissected for in situ electrophysiological studies. Electrodiagnostic evaluation of the RPNI was performed using a 26-gauge stainless steel needle (Natus Medical Inc., San Carlos, CA) placed into the central portion of the RPNI (Figure 2). During both deep and light anesthetic conditions, a painful needle stimulus was delivered to the ipsilateral left plantar skin to induce a withdrawal reflex. A deep plane of anesthesia was confirmed by the absence of reflexes to aural and physical stimuli whereas a light plane of anesthesia was defined as preservation of these reflexes but no volitional movement. Electromyographic (EMG) activity in response to this sensory stimulus was recorded by an electrophysiologic monitoring system (Natus Medical Inc., San Carlos, CA). Nerve conduction studies (NCS) were performed using a stimulating shielded bipolar stainless steel hook electrode (Harvard Apparatus, Holliston, MA) placed around the common peroneal nerve at the level of the sciatic notch. Increasing amounts of current were delivered manually to elicit compound muscle action potentials (CMAPs) until supramaximal stimulation was achieved.

### 2.2. Histology

After testing, animals were euthanized and the RPNI with attached peroneal nerve was removed for histologic examination. Tissue samples were fixed in 10% neural buffered formalin and embedded in paraffin; 5 *μ*m serial sections were subsequently stained with hematoxylin and eosin. Samples were examined under light microscopy at 100x magnification to evaluate the condition and maturation of muscle fibers, axonal regeneration, extracellular matrix deposition, and neovascularization. Separate sections underwent acetylcholinesterase staining to reveal the presence of neuromuscular junctions within the RPNI specimens [10].

Immunohistochemistry was performed on all RPNI samples to determine the presence of myoblast-specific protein desmin within the various scaffolds, demonstrating the survival of the myotubes from implantation to sacrifice. An antidesmin staining protocol involving polyclonal rabbit anti-desmin primary antibody (Thermo Scientific, Waltham, MA) and Cy3-conjugated goat anti-rabbit secondary antibody (Jackson ImmunoResearch Laboratories, Inc., West Grove, PA) was employed with fluorescent microscopy using excitation wavelengths between 515 and 560 nm. An alternative method of visualizing the desmin antibody was to cause a labeled precipitate using 3,3′-diaminobenzidine (ThermoFisher Scientific, Waltham, MA).

### 2.3. Transmission Electron Microscopy

Tissue was immersion fixed in 3% glutaraldehyde in 0.1 M Sorensen's buffer (pH 7.4) and embedded in Epon epoxy resin. Semithin sections were stained with toluidine blue for tissue identification. Selected regions of interest were sectioned into ultrathin slices 70 nm in thickness and poststained with uranyl acetate and lead citrate. These were examined using a Philips CM100 electron microscope at 60 kV. Images were recorded digitally using Hamamatsu ORCA-HR digital camera system operated using AMT software (Advanced Microscopy Techniques Corp., Danvers, MA).

## 3. Results

**Figure 3:**
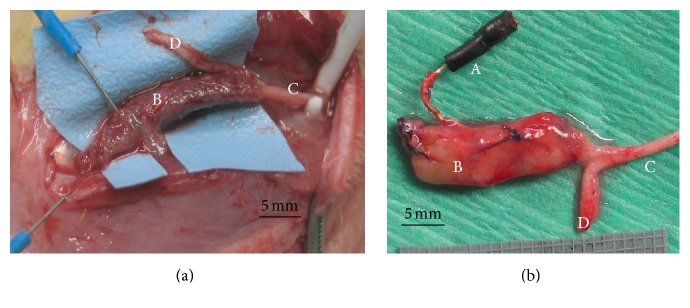
(a) RPNI constructed with silicone mesh scaffold at postoperative day 111. Note that the myotubes that were implanted within the scaffold have matured and the resultant tissue appears pink and well-vascularized. The silicone mesh remained intact and did not negatively affect viability of the surrounding tissues. (b) RPNI constructed with acellular muscle scaffold at postoperative day 270. (A) implanted electrode; (B) RPNI; (C) peroneal branch of sciatic nerve; and (D) tibial branch of the sciatic nerve.

All 25 rats in the study underwent terminal evaluation with electrodiagnostic testing and tissue harvesting. The time from RPNI implantation to euthanasia ranged from 59 days to 111 days with a mean duration of implantation of 93 days. All RPNIs were easily identifiable and clearly vascularized with multiple blood vessels visible on the surface (Figure 3).

### 3.1. Myoblast-Based RPNIs Can Detect Physiologic Efferent Motor Action Potentials When Interfaced with the Peroneal Nerve

**Figure 4:**
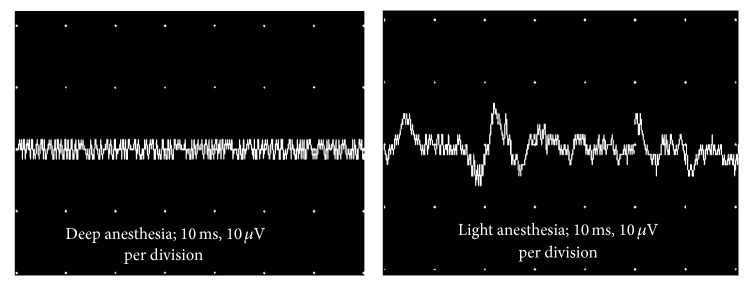
EMG activity recorded from the RPNI in response to painful foot stimulus during deep and light anesthetic conditions.

**Figure 5:**
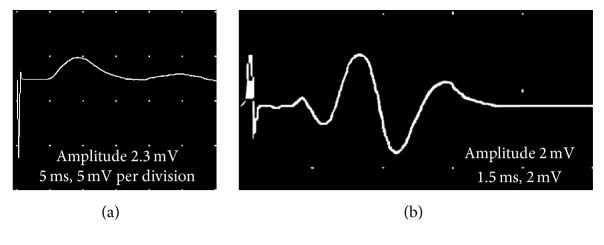
Nerve conduction studies from acellular muscle scaffold RPNI (a) and acellular muscle+PEDOT scaffold RPNI (b) showing the generation of an elicited compound muscle action potential.

EMG activity was recorded from the RPNI during deep and light anesthetic conditions. During a deep plane of anesthesia, a stimulus to the plantar surface of the foot did not result in any EMG recordings. However, when the anesthesia was lightened, distinct EMG signals were directly elicited from the stimulus (Figure 4). Nerve conduction studies demonstrated that CMAPs could be repeatedly produced and recorded through the RPNI in SM, AM, and AM+PEDOT groups with high fidelity and reproducibility (Figure 5).

### 3.2. Myoblast-Based RPNIs Develop into Mature Muscle, Are Reinnervated, Revascularized, and Prevent Neuroma on the End of the Divided Peroneal Nerve

**Figure 6:**
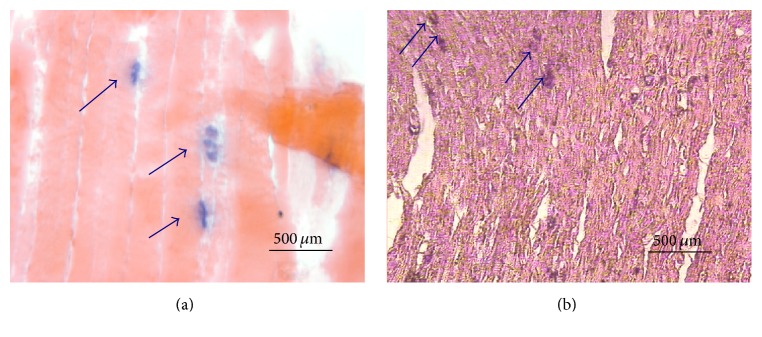
Histologic localization of acetylcholinesterase (arrows) indicates neuromuscular junctions in an acellular muscle scaffold RPNI (a) and an acellular muscle+PEDOT scaffold RPNI (b). Note the formation of neuromuscular junctions in both specimens which suggests successful nerve-muscle synaptogenesis.

Under histologic examination, RPNIs from all groups displayed intact mature muscle fibers and ample numbers of nerve fibers coursing through the substance of the neurotized muscle. Branching blood vessels were readily identified throughout all RPNIs. Occasionally, atrophic or degenerating muscle fibers were recognized, but this was qualitatively similar for all scaffold types. Commonly, groups of regenerated muscle fibers were surrounded by infiltrating connective tissue. Despite the abundance of collagen deposition in all groups, axons were extensively myelinated and no signs of neuromas were detected with light microscopy. In contrast to the SM and AM groups, where axons were identified diffusely throughout the sections, histologic inspection of the AM+PEDOT specimens revealed that regenerating axons were not in close proximity (defined as <1 *μ*m) to the PEDOT material. All specimens displayed the presence of neuromuscular junctions on cholinesterase staining, which suggests the development of motor end plates within the myotube-populated RPNI (Figure 6).

**Figure 7:**
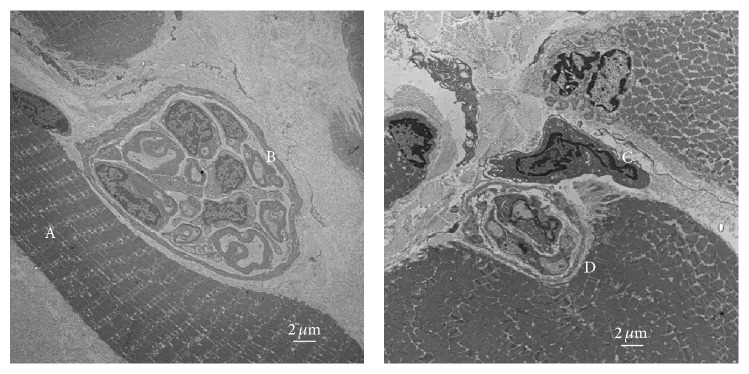
Transmission electron microscopy images of an RPNI created with acellular muscle scaffold at postoperative day 90. Note the presence of mature muscle fibers (A), adjacent regenerating myelinated nerve fibers (B), Schwann cells (C), and the formation of neuromuscular junctions (D).

On TEM imaging, specimens from all three groups displayed ultrastructural evidence of myogenesis, neovascularization, axonal sprouting, and synaptogenesis (Figure 7). Multinucleated cells with organized sarcomeres indicated the successful differentiation and development of myotubes into mature muscle fibers within the RPNI. Vascular channels were discovered throughout all RPNI specimens, even centrally, away from the peripheral initiation of neovascularization. Neuromuscular junctions were found in the samples of all scaffold groups, suggesting that the regenerating axons within the RPNI are capable of nerve-muscle synaptogenesis with myoblast-derived muscle fibers.

**Figure 8:**
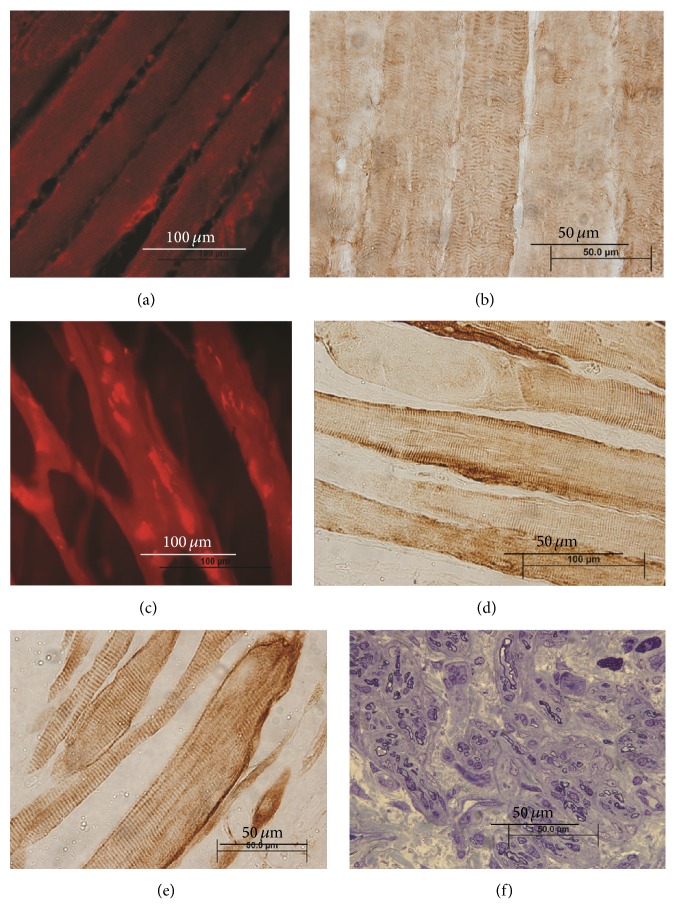
(a–e) Desmin-positive muscle fibers. Desmin staining with striations confirms the survival of myotubes and maturation into muscle fibers after implantation into the RPNI. (a and b) Normal rat muscle fibers. (c) RPNI muscle fibers after maturing inside acellular muscle coated with PEDOT. (d) RPNI muscle fibers after maturing inside acellular muscle. (e) RPNI muscle fibers after maturing inside silicone mesh. Muscle fibers myoblast-derived muscle fibers were found within all RPNI specimens regardless of scaffold material. (f) Example of peroneal nerve cross section at the entrance to the RPNI.

Desmin staining confirmed the survival of myotubes after implantation into the scaffold and supported the premise that the muscle fibers found in the RPNI likely originated from the cultured progenitor cells. Desmin-positive mature muscle fibers were identified in all RPNI samples under fluorescent microscopy regardless of the scaffold material (Figure 8). No observational differences were found in desmin staining between the scaffold groups, suggesting that all experimental environments within the RPNIs possessed adequate tissue perfusion and nutrients to promote myotube differentiation into mature muscle fibers. This was true even in the presence of PEDOT polymer within the substance of the acellular muscle.

## 4. Discussion

High-fidelity control of an upper extremity prosthesis is exceedingly dependent on the crucial interface between the patient and the mechanical device. Several strategies have been proposed to provide this level of prosthetic control. One strategy known as targeted muscle reinnervation involves a surgical procedure which redirects transected peripheral nerves at the site of amputation to proximal muscle groups; upon contraction of these reinnervated muscles, EMG signals can be captured by surface electrodes and used to control a myoelectric prosthesis [11, 12]. To date, this approach provides the most natural, intuitive control for neuroprosthetic devices. Unfortunately, this strategy provides only a limited number of distinct control signals making it difficult to functionally restore many degrees of freedom and permit independent finger, wrist, and elbow motion simultaneously. Other investigators have attempted to provide prosthetic control through direct neural interfacing of the central or peripheral nervous systems [1, 2]. Nevertheless, methods to harness command signals from either the brain or peripheral nerves possess several significant limitations including inadequate signal selectivity and iatrogenic injury to delicate nervous tissue.

The authors present the development of the Regenerative Peripheral Nerve Interface as a new strategy to potentially connect divided peripheral nerves with artificial limbs. As the myoblasts within the RPNI mature into muscle fibers, they are reinnervated by the implanted peroneal nerve, become revascularized, and serve to prevent neuroma formation. In addition, electrical connectivity between the RPNI and peroneal nerve has been demonstrated by CMAPs recorded after both proximal peroneal nerve stimulation and sensory stimulation to the ipsilateral foot. The RPNI displays unique features which address several of the limitations of other experimental peripheral nerve interfaces. First, the RPNI uses contractile myotubes on a biocompatible scaffold in order to generate electrical activity that can be recorded. With reinnervation, relatively low-amplitude nerve signals traveling through the RPNI result in contraction of the myotubes, generating compound muscle action potentials that are readily measured. In essence, the RPNI transduces nerve signals into muscle signals and facilitates detection of efferent motor action potentials. Second, the application of an implanted electrode on the surface of the RPNI construct, distinctly separate from the peripheral nerve, will reduce the possibility of iatrogenic nerve injury either at the time of implantation or due to micromotion over time. Although chronic micromotion may also produce trauma at the interface between the RPNI and the electrode, we posit that muscle tissue will be comparatively more resilient compared to nerve tissue and that much larger muscle action potentials will remain detectable even with expected fibrosis. Third, a separate RPNI can theoretically be interfaced with individual nerve fascicles, allowing for a much greater degree of signal selectivity compared to other available interfaces. Fourth, living myotubes provide regenerating axons a target for reinnervation, thereby reducing the occurrence of misdirected nerve fibers which leads to neuroma formation.

Signals that are transduced through the RPNI must be detected by an electrode in contact with the RPNI. To increase the conductivity between the RPNI and electrode, a biocompatible conductive polymer may be added to the scaffold material. Previous work demonstrated the ability to polymerize PEDOT onto acellular muscle constructs [5, 13]. Ideally, modification of the RPNI scaffold would enhance conductivity without adversely affecting neuronal regeneration, muscle maturation, muscle reinnervation, and neuroma formation. In this study, we found that RPNIs fabricated with AM+PEDOT could sustain viable cultured myoblasts and allow these cells to undergo differentiation into mature muscle fibers once implanted into the rat thigh. Synaptogenesis was also demonstrated histologically in this study group through identification of neuromuscular junctions seen on immunohistochemical staining. Interestingly, while regenerating axons were found adjacent to muscle fibers in all specimens, compared to the SM and AM groups axons were not found in close proximity to the PEDOT substance on histologic examination. While the biocompatibility of PEDOT has been reported by others [14–16], further investigation is required to ascertain the optimum polymerization methods in order to maximize axonal sprouting and muscle reinnervation within the RPNI, while at the same time limiting the potential for neuroma formation.

Painful neuromas can be problematic after upper extremity amputation and multiple surgical approaches have been described to treat symptomatic cases [17, 18]. Many surgeons choose to excise the distal neuroma bulb and implant the nerve into muscle [19]. The RPNI utilizes this concept to address the problem of neuroma in the setting of limb amputation. Current experimental strategies to interface with residual peripheral nerves require application of electrodes around or within nerves which may lead to other unfavorable consequences such as axonal injury and neuroma-in-continuity [20]. Histologic findings of neuroma have been described and include increased myelinated fiber counts and decreased fiber cross-sectional area [21]. Although objective quantification of these specific factors was not performed, we did not find evidence of neuroma formation within any of the RPNIs on either histology or TEM imaging after variable periods of maturation. This observation lends support to the efficacy of muscle implantation as a valid method to treat neuromas and identifies a unique advantage of the RPNI in its role as a possible prosthetic interface.

The authors acknowledge a number of limitations with this proof-of-concept study. Because this experiment represents an attempt to demonstrate the feasibility and potential of the RPNI, the chosen methods were intended to provide a wide variety of information in an expeditious manner, understanding that subsequent work would be required to validate the observations made in this study. Future studies will elaborate on the health of the RPNI by examining the contractile properties of the mature muscle fibers within the RPNI. Nerve conduction studies can clarify the amount of signal that can be transmitted though the RPNI as well as the degree of signal amplification provided by the muscle tissue. Furthermore, harvested tissues can be analyzed quantitatively through histologic techniques to determine the extent of axonal sprouting and synaptogenesis within the RPNI. Despite the subjective nature of this study, our findings encourage the ongoing investigation of this novel peripheral nerve interface.

## 5. Conclusions

This study demonstrates the considerable potential of the RPNI as a novel interface between living peripheral nerves and a neuroprosthetic device. The successful conduction of biologic signals through the use of cultured myotubes within a biocompatible scaffold suggests that perhaps autotransplantation of a unit of muscle may serve a similar purpose. Subsequent studies will explore this possibility and seek to optimize the design such that indwelling electrodes can be chronically implanted with RPNIs for long-term, high-fidelity control of neuroprosthetic devices.
